# COVID-19 Infection in Children and Infants: Current Status on Therapies and Vaccines

**DOI:** 10.3390/children9020249

**Published:** 2022-02-12

**Authors:** Giuseppina Malcangi, Alessio Danilo Inchingolo, Angelo Michele Inchingolo, Fabio Piras, Vito Settanni, Grazia Garofoli, Giulia Palmieri, Sabino Ceci, Assunta Patano, Antonio Mancini, Luigi Vimercati, Damiano Nemore, Arnaldo Scardapane, Biagio Rapone, Alexandra Semjonova, Maria Teresa D’Oria, Luigi Macchia, Ioana Roxana Bordea, Giovanni Migliore, Antonio Scarano, Felice Lorusso, Gianluca Martino Tartaglia, Delia Giovanniello, Ludovica Nucci, Nicola Maggialetti, Antonio Parisi, Marina Di Domenico, Nicola Brienza, Silvio Tafuri, Pasquale Stefanizzi, Luigi Curatoli, Alberto Corriero, Maria Contaldo, Francesco Inchingolo, Gianna Dipalma

**Affiliations:** 1 Department of Interdisciplinary Medicine, University of Bari Aldo Moro, 70124 Bari, Italy; giuseppinamalcangi@libero.it (G.M.); ad.inchingolo@libero.it (A.D.I.); angeloinchingolo@gmail.com (A.M.I.); dott.fabio.piras@gmail.com (F.P.); v.settanni@libero.it (V.S.); graziagarofoli.g@libero.it (G.G.); giuliapalmieri13@gmail.com (G.P.); s.ceci@studenti.uniba.it (S.C.); assuntapatano@gmail.com (A.P.); dr.antonio.mancini@gmail.com (A.M.); luigi.vimercati@uniba.it (L.V.); damianonemore@gmail.com (D.N.); arnaldo.scardapane@uniba.it (A.S.); biagiorapone79@gmail.com (B.R.); dralexandrasemjonova@libero.it (A.S.); mtdoria51@gmail.com (M.T.D.); giannadipalma@tiscali.it (G.D.); 2 Department of Medical and Biological Sciences, University of Udine, Via delle Scienze, 206, 33100 Udine, Italy; 3 Department of Emergency and Organ Transplantation (D.E.T.O.), School and Chair of Allergology and Clinical Immunology, University of Bari Aldo Moro, 70121 Bari, Italy; luigi.macchia@uniba.it; 4 Department of Oral Rehabilitation, Faculty of Dentistry, Iuliu Hațieganu University of Medicine and Pharmacy, 400012 Cluj-Napoca, Romania; 5 University Hospital of Bari, 70124 Bari, Italy; giovanni.migliore@policlinico.ba.it; 6 Department of Innovative Technologies in Medicine and Dentistry, University of Chieti-Pescara, 66100 Chieti, Italy; ascarano@unich.it; 7 UOC Maxillo-Facial Surgery and Dentistry, Department of Biomedical, Surgical and Dental Sciences, School of Dentistry, Fondazione IRCCS Ca Granda, Ospedale Maggiore Policlinico, University of Milan, 20100 Milan, Italy; gianluca.tartaglia@unimi.it; 8 Department of Toracic Surgery, Hospital “San Camillo Forlanini”, 00152 Rome, Italy; giovanniellodelia@gmail.com; 9 Multidisciplinary Department of Medical-Surgical and Dental Specialties, University of Campania Luigi Vanvitelli, Via Luigi de Crecchio, 6, 80138 Naples, Italy; ludovica.nucci@unicampania.it (L.N.); maria.contaldo@unicampania.it (M.C.); 10 Department of Medical Science, Neuroscience and Sensory Organs, University of Bari Aldo Moro, 70124 Bari, Italy; n.maggialetti@gmail.com; 11 Istituto Zooprofilattico Sperimentale della Puglia e della Basilicata, 71121 Foggia, Italy; antonio.parisi@izspb.it; 12 Department of Precision Medicine, University of Campania Luigi Vanvitelli, 80138 Naples, Italy; marina.didomenico@unicampania.it; 13 Unit of Anesthesia and Resuscitation, Department of Emergencies and Organ Transplantations, Aldo Moro University, 70124 Bari, Italy; nicola.brienza@uniba.it (N.B.); alberto.corriero@gmail.com (A.C.); 14 Department of Biomedical Science and Human Oncology, University of Bari, 70124 Bari, Italy; silvio.tafuri@uniba.it (S.T.); pasquale.stefanizzi@uniba.it (P.S.); 15 Department Neurosciences & Sensory Organs & Musculoskeletal System, University of Bari Aldo Moro, 70124 Bari, Italy; lcuratoli@icloud.com

**Keywords:** children, SARS-CoV-2, SARS-CoV-1, COVID-19, antibodies, vaccines, therapy, pregnancy, dentistry, Pfizer

## Abstract

Since the beginning in December 2019, the SARS-CoV-2 outbreak appeared to affect mostly the adult population, sparing the vast majority of children who only showed mild symptoms. The purpose of this investigation is to assess the status on the mechanisms that give children and infants this variation in epidemiology compared to the adult population and its impact on therapies and vaccines that are aimed towards them. A literature review, including in vitro studies, reviews, published guidelines and clinical trials was performed. Clinical trials concerned topics that allowed a descriptive synthesis to be produced. Four underlying mechanisms were found that may play a key role in providing COVID-19 protection in babies. No guidelines are available yet for therapy due to insufficient data; support therapy remains the most used. Only two vaccines are approved by the World Health Organization to be used in children from 12 years of age, and there are currently no efficacy or safety data for children below the age of 12 years. The COVID-19 clinical frame infection is milder in children and adolescents. This section of the population can act as vectors and reservoirs and play a key role in the transmission of the infection; therefore, vaccines are paramount. More evidence is required to guide safely the vaccination campaign.

## 1. Introduction

**Figure 1:**
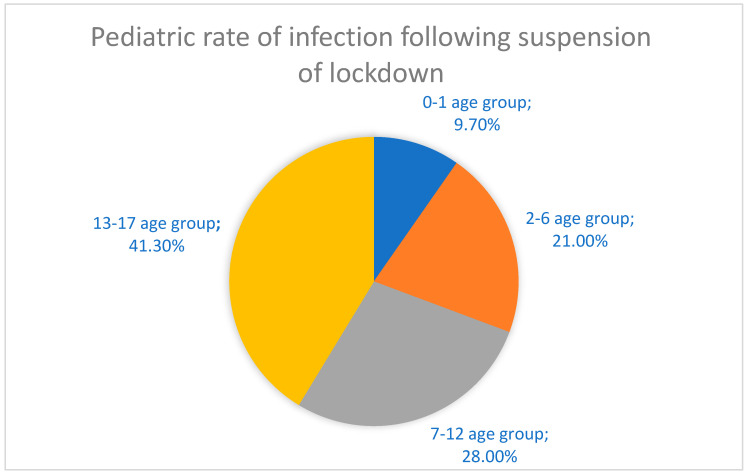
Rate of infection in the pediatric population after 4 May 2020.

The recent global spread of the severe acute respiratory syndrome coronavirus-2 (SARS-CoV-2), which led to the insurgence of coronavirus disease 2019 (COVID-19) has disrupted many human lives and societies all over the world, progressing into a worldwide pandemic that has changed the world permanently [1,2,3,4]. This disease has proven to predominantly clinically affect the adult and elderly populations, and has spared the majority of children, whom, from the beginning, have shown only mild symptoms [5]. The pediatric population percentage that was admitted into the ward for COVID-19 infection was lower compared to the adults’ group although some critical cases have been reported in children as well as in adolescents [6,7,8,9,10,11,12]. Adults and children shared the same cohort of symptoms, which in the latter tend to be milder, leading to a clinical picture close to a common cold that is usually resolved within one or two weeks. Common symptoms are fever, cough, nausea, stomachache, diarrhea, skin rashes, ageusia, anosmia, fatigue, headache, muscular pain, shivers and nasal congestion [13,14]. A study in Nature Medicine, which focused on the transmission patterns of COVID-19 using data from different countries as well as Italy, showed that young people under 20 had a susceptibility to the infection, which can be estimated to be about half that of people aged more than 20 [15,16]. This data have also been confirmed by a multicenter study, involving 82 health bodies across 25 European countries, where evidence reveals that COVID-19 presents itself mostly as a mild disease in children and newborns. When children require admission to intensive care, prolonged ventilatory assistance is needed, generally for 1 week or more, but death is usually not common. The percentage of children admitted to intensive care is 8% [16,17]. Furthermore, data show that coinfection with more viral agents added on top of the infection from SARS-CoV-2 raises the chance that the patient might require intensive care unit support. This knowledge was helpful during the winter period 2020–2021 when there were very few data available and the incidence of other viral infections affecting the respiratory tract, such as the Respiratory Syncytial Virus (RSV) and influenza, were likely to increase [16]. When COVID-19 affects children critically, usually some risk factors are in the background, such as relevant comorbidities, including diabetes, asthma, cardiac congenital diseases, central nervous system diseases and metabolic diseases [14]. To date, information in Italy provided by the latest report of Istituto Superiore di Sanità (ISS) on 7 December 2021, reports a total of 34 deaths in the age group 0–19 of which five deaths are in the age group < 3, five deaths are in the age group 3–5, nine deaths are in the age group 6–11, eight deaths are in the age group 8–15 and seven deaths are in the age group 16–19 [18]. Bellino et al. reported that in the Italian population, by October 2020, pediatric patients represented 1.8% of total infections. Among these patients who were on average 11 years of age and with a slight predominance in male patients, only 13% of patients were hospitalized whilst 3.5% of them needed to be transferred to an intensive care unit. A higher risk of disease severity was associated with preexisting underlying medical conditions [19]. How did children contribute to SARS-CoV-2 transmission? It is important to understand how this happens because children may act as a reservoir [20] for the infection and therefore boost the transmission, especially with the reopening of schools and proximity among many generations [21]. There are limited data available regarding the impact during the first wave, probably because in several countries, including Italy at the beginning of the outbreak, there was an early closure of all educational institutions [15,16]. Closing schools was a common strategy that most countries used in the emergency as a way to try to control the rapid increase in the SARS-CoV-2 infection; this surely helped towards the achievement of this goal but the real impact on the pandemic has not yet been fully detected. A report by ISS that monitored the situation during the period from 4 May to 13 September (transition stage) in terms of the spread of COVID-19 infection tried to evaluate the effect on the scheduled return of activities to normality before the beginning of the school period. Data showed a rise in the frequency of cases reported in both children and adolescents from 1.8%, the percentage reported during the block stage, to 8.5%, the percentage that was achieved during this transition stage [22]. The first wave recorded an epidemic peak at the end of March in patients belonging to the age group < 18 years, whereas the second wave recorded a peak starting from the last week of August and lasting until the second half of September. It is important to underline that asymptomatic cases were primarily observed in the second epidemic wave and this applied to all age groups, including the age group < 18 years. After 4 May 2020, which is when the lockdown was suspended, teenagers belonging to the age group 13–17 had a higher rate of infection (41.3%) compared to the group concerned with children aged between 7 and 12 (28%), those aged 2–6 years (21%) and those aged 0–1 year (9.7%). The patients that required to be admitted to hospital were 4.8%, of which babies aged under 1 year were the most representative (16.2%) (Figure 1). Asymptomatic patients were more than paucisymptomatic (71.2% for the former versus 8.4% for the latter) [22].

Research suggests this increasing rate of infection is due to the reopening of schools. Some data point to the fact that the main issue was failure in complying with prevention regulations, such as the use of face masks, social distancing and hand washing [23,24,25,26]; furthermore, the spread appeared to be more rapid in secondary schools, whereas in preschools, the secondary transmission seemed to be virtually absent [27,28]. A peak of circulation has been reported during the months of December, January and February, which points to a seasonality of coronavirus infection with a lower incidence throughout the year [29]. COVID-19 had a major impact on children’s habits as current data point to the fact that mental disorders in these patients increased in the first year of the pandemic, by 25.2% in children and 20.5% in adolescents; among the symptoms, we enlist depression, anxiety, skepticism, and loneliness, which especially targeted girls and older adolescents [30,31,32]. An increase has also been described in the use of drugs and of behaviors as binge drinking, self-destructive behaviors, pathologic use of social media as well as uncontrolled eating. This important change in eating patterns adjuvated by a decrease in physical activity has led to a growth in obesity rates [33] and overweight conditions in general [34,35]. Another factor was sleep disturbances. Data show that children had trouble falling asleep alone (28%) and therefore expressed a wish to sleep with parents [36].

The current consensus about the therapies to use in children and babies affected by COVID-19 is that in most cases only support therapy is needed, leaving specific pharmacological treatment for more severe cases [37,38]. However, no guidelines are available due to the lack of sufficient data and studies. Vaccines play a key role in preventing and controlling disease infection as they push the immune system to produce antibodies, which aids in the protection against a specific disease [39,40]. Their goal is to contain the spread of the disease by reaching herd immunity and numerous vaccines as of 15 December 2021 have been approved. Vaccines for COVID-19 in children and adolescents carry little knowledge of their efficacy and safety, thus extreme caution is important to assure on one hand, that a proper vaccination is tailored for young patients, on the other hand, that a vaccination campaign for children and adolescents is not delayed as these patients account for one quarter of the world’s population and new variants of concern (VOC) are emerging, such as Omicron, declared on 26 November 2021 by World Health Organization (WHO) [41,42]. The purpose of this investigation is to clarify the mechanism by which children and adolescents in general tend to have this attenuated course of COVID-19 infection and the relevant impact on the therapies, as well as vaccine strategies to adopt for this part of the population.

## 2. Immunopathogenesis

Immunopathogenesis of symptoms induced by SARS-CoV-2 infection is an urgent matter of study. According to the data that have been collected and published throughout these past months of the pandemic, it appears that the immunopathogenesis of the symptoms induced by SARS-CoV-2 infection (generally named COVID or COVID-19) is based on a two-step mechanism [5]: invasion and replication within the respiratory tract and a subsequent viremic phase (responsible for the symptoms [5]). The immune-system from the one induced a “cytokine storm” on the other [43,44,45]. The former appears responsible for the most common symptoms, such as cough, fever, nasal discharge and obstruction resembling diseases as flu, flu-like and the common cold (up to 80% of all SARS-CoV-2 positive patients); the latter instead has been recognized as the molecular basis for the development of the acute respiratory distress syndrome (ARDS), which can lead to hospitalization, non-invasive ventilation (NIV) or even invasive ventilation support and unfortunately, death, as the global community has learnt since the beginning of the pandemic [43]. As new variants of concern emerge and the vaccination campaign has been extended to children in several countries, a deep understanding of how the pediatric section of the global population is affected by and responds to COVID-19 does seem to be a major issue. Both empiric and scientific data show, with a certain grade of reliability, that the younger a patient is, the less likely it is to develop severe symptoms of illness, if positive to SARS-CoV-2 [16,46]. A recent study is trying to correlate this tendency to mainly three different but somehow linked factors: levels of expressions of angiotensin converting enzyme number 2 (ACE-2), preexisting endothelial damage and innate immunity [47]. Higher levels of expression of ACE-2 in children by alveolar epithelium as an age-correlated condition have been correlated with protection from the onset of acute respiratory distress that may arise following sepsis or other non-coronavirus respiratory infections and several other diseases and conditions [47,48]. With regards to the second point in the list, toddlers and young patients can be generally considered as sane and in good health, they have relatively less extended or even practically null endothelial damage in lungs as well as other districts’ micro-circulation, due to the absence of cardiovascular risk factors, such as type two diabetes mellitus, smoking habit, hypertension, which may contribute to avoiding the soaring of the yet cited “cytokine storm”, in a frame of lower inflammatory state of the whole organism [47]. In the end, innate immunity plays a determining role; the recent and constant exposure to the never-encountered-before microorganisms as well as other vaccination cycles than the anti- novel coronavirus’ one may all account for less susceptibility to severe COVID-19 manifestations, i.e., less chance of the “cytokine storm” occurring [47]. Attention must be paid to the fact that newborns represent an exception both at the ACE-2 expression and the innate immunity level, having yet to develop all these systems, being then at a higher risk of hospitalization and severe consequences of SARS-CoV-2 infection [47]. Given this information, it appears to be essential that further studies must be ruled out to truly understand the mechanism that lies behind the SARS-CoV-2 infection.

## 3. Clinical Course of COVID-19 in Children

Literature studies agree that children have a milder course of the disease, and in some cases, are asymptomatic but, in general, pulmonary changes are visible on chest radiographs [49,50]. Children under the age of 12 generally had mild symptoms that resolved within a week, while symptoms in adolescents were similar to those in adults [51,52,53,54,55,56,57]. Regarding the disease manifestation, SARS-CoV-2 infection can lead to asymptomatic cases [58], and COVID-19 ranges from mild flu-like illness (ILI) with mild to moderate development, with a good clinical course [56], to life-threatening complications [59,60,61,62], although pediatric deaths have also rarely been reported [63]. SARS-CoV-2 affects the respiratory tract, resulting in pneumonia, gastrointestinal (GI), cardiovascular or neurological systems [64,65]. There are even less data on MERS-CoV (Middle East Respiratory Syndrome Coronavirus) in children. Most of the documented cases of MERS-CoV were in adults [49]. Manifestations can be severe enough to require intensive care [66].

### 3.1. SARS-CoV-2 and Lung Damage and the Gastrointestinal System

The most frequent mild and moderate clinical pictures involve the respiratory and gastrointestinal systems. In the pediatric population, the most frequently observed symptoms are: fatigue, body aches, headache, low-grade fever, nasal congestion, runny nose, wheezing, dry cough, odynophagia, ageusia and anosmia accompanied by gastrointestinal symptoms, such as stomach pain and diarrhea [19,62,67,68].

### 3.2. SARS-CoV-2 and Acute Multisystem Inflammatory Syndrome (MIS-C)

In children, SARS-CoV-2 may lead to atypical skin manifestations, such as Kawasaki-like disease [69,70,71]. From data reported in the scientific literature, in pediatric and adolescent ages, there seems to be a link between SARS-CoV-2 infection and a rare syndrome, MIS-C Kawasaki-like syndrome, that is similar to Kawasaki disease with other characteristics [72]. MIS-C differs from Kawasaki syndrome by being more severe at onset, by the presence of gastrointestinal symptoms (diarrhea, abdominal pain) or respiratory and pulmonary symptoms (cough, dyspnea) and the presence of inflammatory myopericarditis [73,74]. Furthermore, children with MIS-C are generally older than those with Kawasaki syndrome, which, on the other hand, affects preschool children up to 5–6 years of age [73,74]. MIS-C usually occurs within the fourth week of infection with signs of toxic shock, including elevated inflammatory markers, high fever (38 °C), encephalopathy, conjunctivitis, lips and oral inflammation, redness of the palms of the hands and feet (with or without hard edema and unilateral cervical lymphadenopathy), heart alterations and gastrointestinal complaints [72,73,74,75,76,77]. The correlations between MIS-C and COVID-19 are yet to be defined but the former has some unique characteristics, such as adolescent onset, persistence of abdominal symptoms and numerous cases of left ventricular systolic dysfunction [78,79]. In people under the age of 21, MIS-C shows an incidence of 2:100,000, while SARSCoV-2 infection in the same population during the same period had an incidence of 322:100,000 [80]. MIS-C has been found in the South Asian, Hispanic and African populations. In addition, the detected viral load was low and they had antibodies against SARS-CoV-2 [81,82,83]. The immune system is assumed to be involved in MIS-C expression as it occurs only after the development of antibodies because of infection by the virus. Some studies have reported that the level of antibodies is related to the severity of the infection because they can mediate damage to the system, as there is a production of pro-inflammatory cytokines and chemokines, which activate a pro-inflammatory feedback loop resulting in barrier damage alveolo-capillary [84,85]. In this syndrome, as in Kawasaki disease, genetic alterations of the immune system are present and put children at greater risk by modulating the responses of T and B lymphocytes [86]. Moreover, those children have a more active immune response and have healthier airways because they have not been exposed to too much cigarette smoke and air pollution as adults have [87,88]. Patients with severe MIS-C have a high dose of neutrophils, D-dimers, C-reactive protein, lactate dehydrogenase, ferritin, troponin and natriuretic peptide B compared to patients without such manifestations [81]. Furthermore, they have lymphopenia, anemia, hypoalbuminemia and coagulation defects [81]. Prolonged fever, together with high systemic inflammation and some characteristics of Kawasaki disease (atypical form) are at the basis of a possible new spectrum of vasculitis [82,83].

### 3.3. SARS-CoV-2 and the Cardiovascular System

Cardiac changes and coronary aneurysms have been found to appear in 10–20% of cases and mortality in 2–4% of patients [81,82,83]. A study conducted at Wuhan Children’s Hospital collected data on 157 hospitalized pediatric patients diagnosed with SARS-CoV-2 [89]. Of 157, 148 patients between 18 months and 10 years of age showed mild or moderate disease [89]. On laboratory examination, they showed high levels of alanine aminotransferase (ALT), aspartate aminotransferase (AST), creatine kinase MB (CK-MB) activity and lactate dehydrogenase (LDH), suggesting liver and cardiac injury [47,90]. Some cardiovascular events have been reported since the earliest cases of COVID-19, including myocarditis [91,92]. The remarkably high rates of IgG and IgA identification strongly suggest a post-viral immunological reaction impacting the myocardium [93]. The underlying mechanism of heart damage remains unclear as none of the patients had an endomyocardial biopsy [78]. Moreover, the dramatic cardiac function improvement as well as the significant decrease of inflammatory biomarkers following intravenous immunoglobulin reinforces the hypothesis of a SARS-CoV-2 post-infective disease [93]. Careful follow-up of cardiac recovery and repeated ultrasound scans are required to assess the possible onset of coronary artery dilation [78]. Involvement of the myocardium and direct damage to vital organs has also been reported in adults [94,95].

### 3.4. SARS-CoV-2 and the Neurological System

SARS-CoV-2 in children < 18 years frequently causes neurological complications, such as headache (4%), anosmia (2%), convulsions (0.7%) and cerebrovascular stroke (0.7%) [96,97]. The pathophysiology of neurological complications can be multifactorial: viral replication in the CNS, circulatory disorders caused by vasoconstriction and/or occlusion, non-specific complications of MIS-C and altered inflammatory response [98,99,100].

## 4. COVID-19 Protection in Newly Born and Children

**Figure 2:**
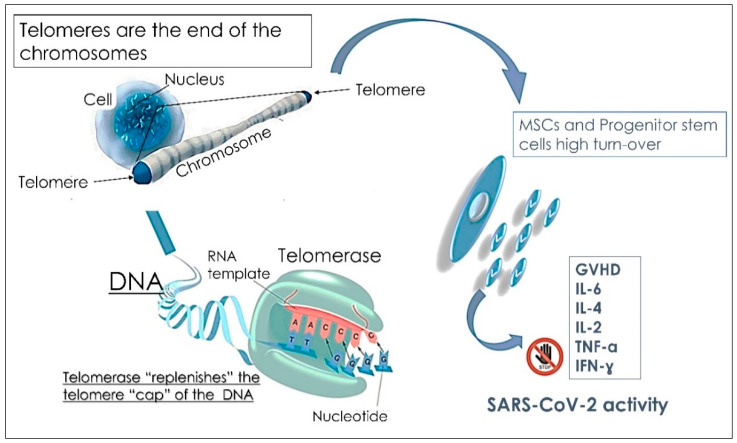
Schematic representation of telomeres as a key factor in sustaining MSCs and progenitor stem cell proliferation, thus leading to their anti-inflammatory effect by blocking the production of pro-inflammatory cytokines (IL-6, TNF-α, IFN-γ). [interleukine: IL; tumor necrosis factor-α: TNF-α; Interferon gamma: IFN-γ; graft versus host disease: GVHD].

One of the most intriguing questions about the new SARS-CoV-2 is why children are less infected than adults. Many mechanisms to explain this have been explored [101]. The first putative mechanism involves the receptor expressed on alveolar type 2 cells: the enzyme of conversion of angiotensin 2 (ACE-2) that SARS-CoV-2, as well as the human coronavirus NL63 (HCoV-NL63), infect human cells. Indeed, the distribution of this receptor may be different and may differ based on children’s age [102]. Many authors have previously reported that the different distribution, maturation and activity of ACE-2 receptor may explain the lower incidence of SARS-CoV-2 in children [16], suggesting that a lower expression of ACE-2 in children’s lungs may reduce SARS-CoV-2 infection and, hence, COVID-19 clinical manifestation in children. In rats lungs, it has been described that a reduction of ACE-2 expression occurs as it ages and that ACE-2 receptor protect lungs from lesions, sepsis and virus infection, including SARS and the avian influenza A H5N1 [46]. Infants less than 1 year old have the highest risk of clinical complications, and they should have a lower expression of ACE-2 receptor that may not protect children from clinical complications. For these reasons, SARS-CoV-2 and/or coinfection with other viruses or bacteria should be treated and carefully monitored. Another mechanism involves the endothelial damage that increases in line with cardiovascular diseases, diabetes mellitus and tobacco smoke and that are risks factor for severe COVID-19. Accordingly, pre-existing damage in the endothelium may foster an inflammatory response to SARS-CoV-2 and increase inflammation [101,103,104]. By contrast, the endothelium of healthy newborn are not damaged, thus avoiding exacerbating the anti-SARS-CoV-2 response. Moreover, the third hypothesis that may explain the lower susceptibility of children to COVID-19 regards the first line of defense against SARS-CoV-2: the innate immunity. The main immunoglobulins involved in the response to SARS-CoV-2 are IgM, IgG and IGA. Analysis of anti-SARS-CoV-2 IgG, IgM and IgA may play a complementary role in assessing the immune status of individuals [105,106]. Anti-SARS-CoV-2 antibodies develop three days after the onset of symptoms or one week after SARS-CoV-2 infection. Notably, IgM antibody concentration peaked before IgG antibody production. Specific IgM is the early antibody response that begins and peaks within 7 to 12 days and declines significantly after 18 days; in contrast, specific IgG antibodies develop a few days later (10 to 18 days), do not decline, and persist throughout several months as protective antibodies [107]. A high and persistent level of antibodies to SARS-CoV-2, and in particular neutralizing antibodies to RDB-IgG, which recognize the spike protein of SARS-CoV-2 and prevents infection of cells, represents a strong indication that an immunized host may resist infection by SARS-CoV-2. Neutralizing antibodies are able to block a pathogen from infecting the body by inhibiting molecules on the surface of the pathogen used to enter cells [108]. Neutralizing antibodies result in permanent immunity to SARS-CoV-2 infections. In fact, the detection of anti-SARS-CoV-2 antibodies can be used as a passport to immunity or as evidence of prior infection or immunization. Studies have shown that COVID-19 patients with high IgG titers produce antibodies with neutralizing activity that can destroy the virus. There is a correlation between IgG levels of the anti-SARS-CoV-2 spike protein and neutralizing antibody levels in COVID-19 patient plasma [109]. Most serological tests focus on IgM and IgG antibodies, although IgA antibodies play an important role in mucosal immunity and represent the most important immunoglobulins involved in the immune response against pathogens of the respiratory and digestive systems [110,111]. The development of mucosal immunity through IgA may be important in the prevention of SARS-CoV-2 infections [112]. Indeed, the virus recognizes and infects respiratory epithelial cells by binding to the ACE-2 (angiotensin-converting enzyme-2) protein on the surface of alveolar type 2 cells [113]. Furthermore, in addition to typical respiratory symptoms, digestive symptoms, including nausea, vomiting, diarrhea, and anorexia, may occur in patients with COVID-19. Some patients may develop digestive symptoms in the absence of respiratory symptoms [114]. Therefore, IgA testing might be useful, along with IgG and IgM, to monitor and recognize patients with atypical symptoms and in paucisymptomatic cases (including mild conjunctivitis, low fever and digestive symptoms) or in suspected individuals with a negative reverse transcriptase-polymerase chain reaction (RT-PCR) result for a nasopharyngeal swab [115]. To this end, the anti-SARS-CoV-2 humoral response may support and improve the diagnosis of COVID-19, including subclinical and asymptomatic cases. IgA production is peculiar and, initially, IgM kinetics are parallel: IgA and IgM levels increased 6–8 days from the onset of symptoms, then IgA showed persistently higher levels for 38 days, peaking on days 20–22, while IgM levels peaked on days 10–12 and decreased significantly by day 18 [116]. Coronaviruses are able to evade innate immunity by blocking interferon type I cascade. Viral infection, as well as vaccines that are administered to infants from the first months of their life, stimulate innate response in children [113,117]. Thus, the frequent vaccinations that newborns undergo may support innate immunity against SARS-CoV-2. In line with this hypothesis, the importance of RNA vaccine administration should be considered, including the influenza vaccine that also uses the route of interferon 1, to support the immune response in adults [118,119]. For this reason influenza vaccine administration may prevent SARS-CoV-2 infections [118,119]. Furthermore, the lower maturity and function of ACE-2 receptor in children compared to adults may halt virus infection of host cells [120]. In addition, as children still have an immature immune system, they respond to infections via different mechanisms thus overcoming SARS-CoV-2 infections [120]. Some authors suggest that innate immunity plays a pivotal role in the protection against the new coronavirus [119,121]. When a virus infects the host and invades cells, the immune system of the host activates several signals to prevent viral infection and slow their replication, including the production of interferon type 1, the activation of natural killer cells and the Toll-like receptors (TLR) [121]. After that, activation of CD8+ T cytotoxic lymphocytes occurs and neutralizing antibodies are released, thus preventing the virus in the extracellular environment from invading other cells [119]. Furthermore, the RNA positive single stranded virus, the TLR7, 8 and 9, and the adaptor of myeloid differentiation primary response 88 signal (MyD88) activate NF-kB and interferon production. NF-kb activation triggers the cascade of inflammatory cytokines [113,121]. This complex mechanism may be functional against virus infection without the activation of adaptive immunity. Of particular note, the innate immunity is fully functional during the first years of life [122]. The immune system activates adaptive immunity after the first years of life and continues to function and improve for the following 10 years. A complete innate immunity response is reached after the first six months of life [113]. A comparative study between 32 adults and 47 children showed that children mainly released antibodies against the spike protein of SARS-CoV-2, the protein the virus used to invade cells. By contrast, adults produced a different set of antibodies, including the ones against the spike protein as well as antibodies against the nucleocapsid protein, necessary for viral replication, which are released in the whole body [123]. The ability to eradicate the virus in children may be due to their competent innate immune response [124]. Indeed, children do not display antibodies against nucleocapsid, suggesting that viruses do not infect the whole host and do not replicate. Thus, the immune response of the newly born seems to be able to eliminate the virus before its replication occurs. Furthermore, children may be less infected as they are socially isolated compared to adults, thus they are less exposed to SARS-CoV-2 infection. In addition, they also have a reduced exposure to smoke, and have an increased lung regeneration capability [87,88,125]. Neutrophils of adult patients form extracellular nets that may damage organs and may lead to the high mortality of COVID-19. However, the formation of these extracellular traps in children still does not occur [122]. Children have a young immune system, while adults have an exhausted immune system that may lead to chronic inflammation [88,126]. Mesenchymal stem cells (MSCs) represent a great potential against infections. In young people, MSCs are highly proliferating cells and have an increased turnover [127]. Children have a mild differentiation of MSCs, and several factors are involved in cell differentiation and in immune response, including proliferation of progenitor cells, skeletal muscle metabolism and angiogenesis. Furthermore, the presence of long telomere and the presence of still immature ACE-2 receptors, do not allow SARV-Cov2 to infect the cells of a young host as well as allow virus replication and invasion [128]. Telomere shortening occurs during any cell division and it is crucial during acute and chronic inflammation, oxidative stress and other conditions that trigger cell proliferation, tissue repair and immune response [128,129]. Thus, the regenerative process, which is very active in young people, may represent one of the main processes that protect them from SARV-CoV2 infection and COVID-19 symptoms [127]. Finally, the fourth hypothesis suggests that the role of MSCs in the treatment of several diseases appears to be critical in reducing the inflammatory processes of respiratory infections caused by COVID-19. In pediatric subjects, the internal mucosa, tissues and epithelial cells of the lungs can be potentially damaged by frequent respiratory infections by constantly activating the presence of surveillance immune cells [127,130]. In this scenario, local MSCs interact with the local immune system, inducing the activation of specific immune cells, such as gamma and delta T lymphocytes (γ/δ T Cells) and macrophages type 2 (M2). Furthermore, almost all MSCs were able to differentiate into alveolar type II (ATII) epithelial cells and active repair signaling pathways. MSCs represent 60% of the pulmonary alveolar epithelium [46,131]. In children, these events rarely take place, and this is probably due to the regenerative patterns typical of this age. The high number of MSCs induce an immediate response that prevent the release of pro-inflammatory cytokines and interleukins, such as TNF-α, IFN-γ, IL-6, IL-2 and IL-1. The anti-inflammatory activities of MSCs take place in two steps: the first one involves the ability of MSCs to influence monocyte differentiation towards dendritic cells (DCs) and M2 and, the second one by inducing tolerant phenotypes of naive and effector T cells, by blocking antibody release from B cells and also by suppressing NK cell proliferation and NK cell-mediated cytotoxicity [127,132]. The strong regenerative potential as well as the long telomeres and the immaturity of ACE-2 receptors of young people prevent SARS-CoV-2 infection Moreover, the high proliferation rate of MSCs and their immunomodulatory activity counteract virus dissemination [127]. The high number of MSCs and the fast turn-over specifically seen in children might provide an explanation of their immunity to SARS-CoV-2. The fast tropism of SARS-CoV-2 would be the major weakness underlying this claim that it tends to lose efficacy whenever it needs to replicate as a result of the elevated presence of a strong immunomodulatory environment [132,133]. The reduced ability of adults and elderly patients to respond to COVID-19 treatment may be due to their restricted self-renewing capacity of circulatory MSCs [134,135]. Throughout each stem cell division, the shortening of every single cell telomere occurs [128]. Telomeres are specific DNA-protein arrangements that firmly seal the ends of linear chromosomes. The telomeres require a constant extension of telomeric DNA repeats to maintain the chromosomes’ stability [129]. Consequently, telomere modification may lead to radical chromosome alteration and to a systemic effect [134]. Furthermore, the reduction of telomere length is further intensified during both chronic and acute inflammation as well as in oxidative stress condition, since these mechanisms trigger cell division to ensure tissue repair and immunological reaction. Consequently, telomere reduction indicates an exhausted replicative ability that strongly contributes to a decreased resistance against infection [134]. As telomere length and age are influenced by several factors [135], the regenerative process in children may justify their protection against SARS-CoV-2 (Figure 2) [135]. Therefore, there are two mechanisms which may protect children from COVID-19: (i) the high amount of circulatory MSCs and of progenitor stem cells, and their strong regenerative potential; and (ii) the telomere length that ensures the fast replication of stem cells, and their expansion [136]. In conclusion, children have a high regenerative process due to the elevated rate of circulating stem cells and for the activation of an adaptive immune response [136].

## 5. Variants

An important number of SARS-CoV-2 variants have evolved after the epidemic began in China in December of 2019 [1,2,137]. Viruses spread by frequently mutating or altering their initial composition. A variant is a duplicate that varies from the primary virus [138]. Throughout the time of the pandemic, the SARS-CoV-2 primary version has mutated several times, then when detected, the WHO listed every one of the new coronavirus variants with a letter that belongs to the Greek alphabet [138]. The main differences between a primary virus and a variant lie in the possibility to get reinfected, the reaction to treatments, in what way the patients infected evolve and the possibility of transmission [138]. Until this moment, the WHO defined the VOC as a variant with enhanced infectiousness, virulence, and with a low rate of reaction to current treatment [139]. On 18 December 2020, designation was given to the first major variant that was documented in September in the United Kingdom (UK). This variant received the name Alpha, with the Pango lineage B.1.1.7. This was considered as a VOC because of its increased transmissibility and the potential immune evasion that consists of seven missense mutations (N501Y, A570D, D614G, P681H, T716I, S982A and D1118H) and also a number of three deletions in spike protein (69/70del and 144del) [139,140,141]. The spike protein is formed from two subunits: the first one is named S1 and it carries the receptor binding domain (RBD), and the other one S2, that has a main property of the viral–host cell merging. The mutation that is mainly encountered in the genome of the virus is the replacement of D614G at the level of S1, by this replacement the connection of the virus to the ACE-2 receptor is expanded, determining enlarged host sensitivity and enlarged transmission [139,142,143,144]. The second major variant, that was documented also in 2020, was the Beta or with the Pango lineage B.1.351 (501Y.V2) variant that appeared in South Africa in May 2020 [142]. The third variant is known by the label Gamma or the so called pedigree P.1 (Japan/Brazil) and the fourth variant was named Delta or pedigree B.1.617.2 (India) [139,145].

When compared to prior versions, the Omicron version appears to increase by 2.4 times the probability of re-infection for the patients that have passed through the infection. This aspect is probably owing to the significant number of mutations present in comparison to prior versions. The variant B.1.1.529 (Omicron) spread very fast compared to the Delta variant [146]. Studies regarding the extent of protection conferred by different vaccines for the Omicron form are still pending [147,148]. An analysis published by Reuters reveals the presence of eight main strains of SARS-CoV-2 after the evaluation of 185,000 genomes. These include the following strains: Strain L, S, V, G with subgroups GR, GH and GV, VUI 202012/01 and Strain O [143].

## 6. Therapy

The Italian Society of Pediatric Infectious Diseases (SiTIP) performed a literature review on recommended treatments in children with COVID in which it was noted that most of them have a benign clinical course. Therefore, it was recognized that pharmacological treatment other than adjuvant therapy should be reserved for more severe cases [37]. Due to the fact that there are insufficient data on therapy for COVID-19 in neonates, guidelines have not been created and thus treatments are not standardized [149]. Treatment should be modulated according to the clinical course (Table 1) [150].

MIS-C correlates with many clinical cases [82]. The treatment of cases without symptoms is not required; in mild and moderate cases it is advisable to use paracetamol; in severe and critical cases, antibiotic, antiviral and immunomodulatory therapy is administered as appropriate [37]. In cases of obstruction, airway aspiration is performed. In addition to this, oxygen therapy using nasal cannula or face masks, hydration and diuresis monitoring is carried out (Table 2).

## 7. Antipyretic Therapy

Paracetamol is administered for fever > 38 °C (10–15 mg/kg every 4–6 h). Ibuprofen is not advised in case of dewatering, diarrhea and vomiting because it may lead to the likelihood of kidney failure [151].

## 8. Lactoferrin

Lactoferrin contained in large quantities in breast milk is a protein contained in serum that has several biological properties, including that of metabolizing iron by binding to it and releasing it. In addition, it controls the immune response versus viruses, bacteria and fungi: it operates on cell receptors, blocking viral attachment and adhesion to the surface of host cells, suppressing the entry of the virus inside the cell [154]. Iron homeostasis and inflammation are governed by it. In fact, high levels of IL-6 are shown in the disorder of iron homeostasis [155,156]. This protein in colostrum has a peak concentration of 8 mg/mL, in mature milk it is 3.5–4 mg/mL and in exocrine secretions and mature neutrophil granules it is even lower. The concentration increases in cases of inflammation and/or infection as neutrophils are present [155,156,157]. It has been shown that giving milk with high lactoferrin concentrations in premature infants decreases the risk of respiratory and intestinal sepsis [158,159]. Viruses, particularly coronaviruses, identify cellular anchoring sites in the first phase of the infectious process. Lactoferrin operates with cellular receptors and is identified in heparan sulphate proteoglycans (HSPG) [160]. Lactoferrin has been found to inhibit infection because it hides the virus anchoring sites provided by HSPG by preventing adhesion between host cells and SARS-CoV-2. Lactoferrin’s blockade of the binding between HSPG and viral spike protein differs from that on the ACE-2 receptor (Table 3) [161].

After initial binding, the virus seeks out other receptors for entry into the host cell. Lactoferrin therefore prevents subsequent steps, such as quantity of virus on the cell area and the identification of entry receptors, such as ACE-2 [160,162]. There are several studies on these mechanisms with SARS-CoV-1 but not SARS-CoV-2, even though between the two types of coronavirus there is a 72% equal genome sequence, thus the receptor binding is very similar; these data are important [154,163,164]. Many studies show that SARS-CoV-1 spreads via breath droplets but can also infect enterocytes causing gastroenteritis and acting as a carrier. The main clinical signs in infants are gastroenterological in nature [165,166,167,168]. Whey, lactoferrin and milk in the first few months build a favorable gut microbiota by enhancing an innate immune defense in infants [157].

## 9. Aerosol

As aerosol would increase virus transmission. The use of nebulizers is recommended to avoid this: inhalation therapy includes the use of a bronchodilator and/or a topical steroid in the event of wheezing [169,170]. If there is a fever lasting more than 3 days, there may be a bacterial infection and antibiotic therapy may be associated with it, otherwise it should be avoided [171]. Vital signs should be checked every 8 h or depending on the clinical picture [171]. It is important to assess the usage of immunomodulators, such as interleukin inhibitors methylprednisolone, if possible, such as Anakinra or Tocilizumab. If there is a worsening of lung function, their use is recommended 7 days after the onset of symptoms and in case of increased IL-6 and/or D-dimer and/or ferritin and/or C-reactive protein [172]. Prevention of thromboembolic events as a complication in children, although having a lower incidence, is possible with heparin; however, this treatment is usually not provided in children [173]. This could be regarded for infants and adolescents where manifestations of thrombi are more usual [174]. The treatment prescribed is subcutaneous enoxaparin 100–200 U/kg/day, which may be enhanced to 150–300 U/kg/day in neonates (Table 4).

## 10. Antiviral Treatment

This is only implemented for children in severe or critical condition, and should be used as part of a clinical trial [150].

### 10.1. Lopinavir/Ritonavir

This is the most recommended antiviral (Table 5), and is given after the first 14 days of life, as supportive therapy to other human immunodeficiency virus (HIV) drugs [175]. It cannot be used in premature infants born before 42 weeks [152].

### 10.2. Remdesivir

Is an investigational drug (Table 5) recommended for the treatment of COVID-19 in children [37,152]. It has been shown that if combined with hydroxychloroquine sulphate or chloroquine sulphate, it may have a lesser pharmacological effect [176].

### 10.3. Hydroxychloroquine

The antiviral mechanism of action of hydroxychloroquine and chloroquine is still unclear [177]. Hydroxychloroquine has greater results in vitro compared to vivo. In addition, it is unclear whether the benefits outweigh the dangers, such as ventricular tachycardia and QT interval prolongation. The danger increases when associated with certain antibiotics, such as azithromycin (Table 6). Therefore, other studies are needed to validate the usage in children with COVID-19 [153,178].

An electrocardiogram (ECG) should be performed prior to use to rule out long QT and to assay glucose-6-phosphate dehydrogenase (G6PDH) prior to use.

## 11. Immunomodulatory Therapy

It is carried out in case of ARDS, worsening of respiratory activity and MIS, a rise in ferritin and/or C-reactive protein and/or IL-6 and/or D-dimer [172]. It may only be administered if there is pulmonary worsening for a least 7 days after the startI of symptoms (Table 7) [172].

## 12. Antibiotic Therapy

In patients without co-morbidities, the antibiotics to be administered (when fever lasts more than 3 days [179] are as follows:

- Amoxicillin: 90 mg/kg/day in 3 doses, when administered per day.
- Ceftriaxone: 80–100 mg/kg/day, if oral administration is not possible. This drug reduces the risks of exposure and transmission between operators as it can be administered once a day.
- Azithromycin: 15 mg/kg on the first day, then 7.5 mg/kg once a day for a further 4 days.

The lung damage caused by COVID-19 is due to an attack on pneumocytes II and I. This leads to a cascade of cytokines (including IL-6) causing micronecrosis and secondary lung necrosis. This drug acts against both soluble and membrane IL-6 receptors [180,181]. Therefore, in addition to being an excellent drug against COVID-19, it is already given to children for the therapy of rheumatoid arthritis, juvenile idiopathic polyarthritis (for 2 years), systemic juvenile idiopathic arthritis (for 1 year) and Cytokine release syndrome (CRS) (for 2 years) [181].

The dosage of Tocilizumab 20 mg/mL vials is as follows:

1. First infusion: 10–12 mg/kg < 30 kg and 8 mg/kg > 30 kg (maximum dosage 800 mg, infusion d15or15ox.n approx. 60 min);
2. Second infusion: 12 h after first infusion (according to the advice of doctor, in case of no response).
3. A third infusion can also be considered after 24 h.

The plasma dosage is rated for IL-6 and/or D-dimer one day after the last injection [181]. Several studies show that most SARS-CoV-2 infections have a favorable course in children, so drug treatment without adjuvant therapy should only be proposed in severe cases [47,150,182].

## 13. Probiotic Therapy

Many studies claim that probiotics can be an excellent defensive weapon against SARS-CoV-2 infection as well as for other diseases, such as cancer and diabetes, leading to better clinical outcomes. They have been shown to be effective in lowering inflammation both by inhibiting inflammatory interleukins and by raising antibody levels [183,184].

## 14. Vaccines

**Figure 3:**
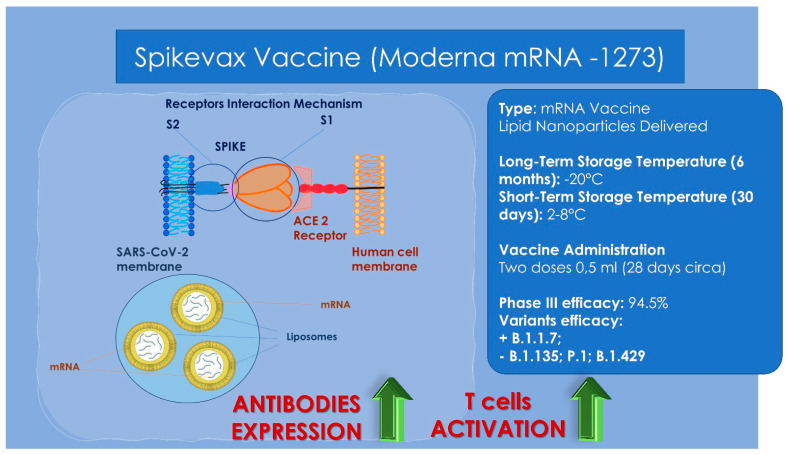
Moderna vaccine general characteristics, storage properties, administration dosages and variants’ efficacy. High: the receptors key-to-enter interaction mechanisms between the viral vector and the human host cell membrane. Low: the lipid-based nanoparticle with the mRNA is able to enter through a cellular endosome into the human cell. Inside the cell, the ionizable lipidic component of the endosome membrane is able to become charged positively producing a release of the lipid-based nanoparticle and mRNA into the cytoplasm. Then the ribosomes is able to synthetize proteins and messengers from mRNA producing an increase of the antibodies’ expression and an activation of the T cells’ response.

**Figure 4:**
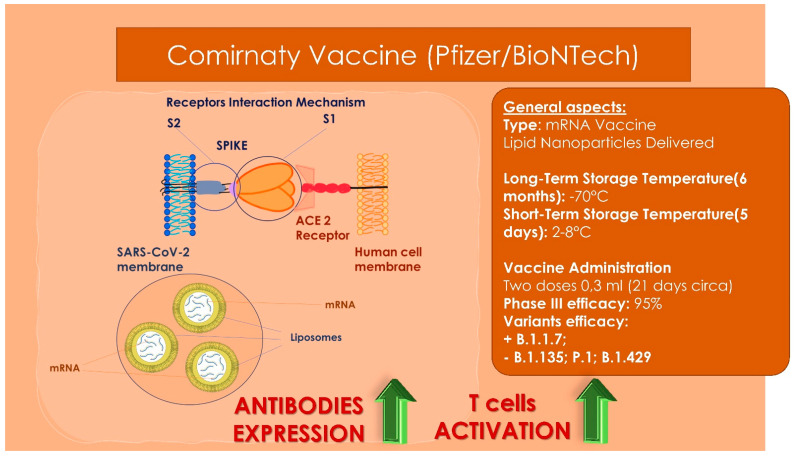
Pfizer/BioNTech vaccine general characteristics, storage properties, administration dosages and variants’ efficacy. High: the receptors key-to-enter interaction mechanisms between the viral vector and the human host cell membrane Lower: the lipid-based nanoparticle with the mRNA is able to enter through a cellular endosome into the human cell. Inside the cell, the ionizable lipidic component of the endosome membrane is able to become charged positively producing a release of the lipid-based nanoparticle and mRNA into the cytoplasm. Then the ribosomes is able to synthetize proteins and messengers from mRNA producing an increase of the antibodies’ expression and an activation of the T cells’ response.

Pre-existing medical conditions clearly predispose children to severe disease, but healthy children are also at risk for severe COVID-19 [7]. A recent study carried out in two states of South India, Tamil Nadu and Andhra Pradesh, has demonstrated transmission among children in childcare facilities, schools, summer camps and at the population level [185]. In addition to the direct health benefits of active immunity to SARS-CoV-2, a safe and effective pediatric vaccine could dramatically mitigate the marked social impact of COVID-19 upon children [186]. Pediatric COVID-19 vaccines could also restore other experiences that have intangible benefits upon children (e.g., extracurricular activities) [187]. Vaccines, as an effective way to prevent and control disease infections, stimulate the human immune system to produce antibodies, thus increasing immunity to the disease and generating protection for the immunized individual [39,188]. The first reason for vaccinating children is to protect them from illness [189]. While most of the children diagnosed with COVID-19 have only mild symptoms—even asymptomatic in nearly half of the cases—since the start of the pandemic, 399,000 cases were found in children under nine years, which led to 3398 hospitalizations, 177 intensive care unit (ICU) admissions and 20 deaths [189]. The second reason is that infections in the general population can be prevented by vaccinating children [189,190]. Only two RCTs (randomized controlled trial) on children and adolescents have been published in peer-reviewed journals, both of which found that the respective vaccines, BNT162b2 and CoronaVac, are safe and effective (Table 8). Clinical trials on vaccines are currently underway but not yet completed [39]. Both Moderna (Figure 3) and Pfizer (Figure 4) vaccines are licensed for use in children over the age of 12. Adolescents aged 12 to 17 years with related diseases have an increased risk of severe COVID-19, so other high-risk groups can be vaccinated to protect them [191]. For the time being, there is no scientific evidence on safety and efficacy in children under 12 years of age [191]. Until there are sufficient data, this group should not normally receive the vaccine [191]. China has nevertheless extended the authorization of the Pfizer-BioNTech/Comirnaty COVID-19 vaccine for use in children aged between five and 11, with inoculations set to begin before the end of the year [192]. The Singaporean Minister of Health (MOH) announced that the multi-ministerial task force has agreed to the pediatric use of the Pfizer-BioNTech/Comirnaty vaccine in the 5–11 age group, according to the COVID-19 Expert Committee on Vaccination [192]. The emergency use of BNT162b2 by Pfizer (Figure 3) and mRNA 1273 by Moderna (Figure 4) has been authorized in the population between 12 and 17 years of age [193]. Furthermore, in November 2021, BNT162b2 vaccine was also approved in children of 5–11 years [193]. Amongst the side effects of mRNA COVID-19 vaccine in children, pericarditis and myocarditis have been reported; even if these complications are mild, responsive to conservative treatment and less severe compared to classic or COVID-19 related myocarditis [193]. Available data report that male children were more affected from myocarditis and pericarditis following vaccination [39]. Schauer et al. referred to a 0.008% incidence of myopericarditis in adolescents aged 16–17 and of 0.01% between 12 and 15 years of age after the second dose [194]. However the risk of myopericarditis following vaccination is lower than that related with SARS-CoV-2 infection [195]. In October 2021, the Global Advisory Committee on Vaccine Safety (GACVS) stated that deaths and severe COVID-19 disease are significantly reduced by the introduction of COVID-19 vaccines at all ages so the benefits of vaccines overcome the risks [193]. It is not possible to asses with certainty whether vaccines should be mandatory in children as there is still little knowledge about their efficacy and the epidemiology of COVID-19 in this age group [39]. For this reason, the risks and benefits of vaccines in children and adolescents should be carefully studied and monitored [39]. In conclusion vaccination of children and adolescents represents an important step towards reaching herd immunity but further studies are advised to assess risk–benefit balance and evade possible complications of a rushed vaccination campaign. The major scientific societies in the world recommend vaccination to those who are pregnant and breastfeeding. Vaccinating pregnant women offers an opportunity to provide protection to immunologically immature infants before they can receive their own vaccinations [196]. There is no certainty of pregnancy interruption in women who received vaccination before discovering they were already pregnant [47]. Antibodies found in women’s breast milk showed strong neutralizing effects, suggesting a potential protective effect against infection in the newborn [96]. Thus vaccination is not contraindicated in breastfeeding women [47]. There are consistent data to validate that vaccination during pregnancy and breastfeeding causes higher antibody responses than infection [47].

## 15. Discussion

Different pathways explain why children and adolescents seem to have a boost in the protection towards COVID-19 compared to adults. More studies are required to confirm these hypotheses, especially because some could also be exploited in the design of a tool to be used in certain subsets of patients, for example, mesenchymal cells in immunocompromised patients [132,139]. Among the various therapeutic options, no treatment is recommended for asymptomatic individuals, but in children with mild or moderate symptoms, the use of antipyretics is recommended. In severe and critical cases (ARDS or MIS), anti-inflammatory drugs such as corticosteroids, antibiotics, antivirals such as Remdesivir or hydroxychloroquine, immunomodulators such as Anakinra and monoclonal antibodies such as Tocilizumab, are recommended. Probiotics or milk with high lactoferrin concentrations can be administered as adjuvant therapy to boost the immune system [37]. Vaccines for SARS-CoV-2 in children are an important topic for their own safety as well as for their possibility of being vectors and reservoirs in spreading the infection [20]. Discordant recommendations are available at the present time due to insufficient data and not enough studies to prove the safety of vaccines in specific group ages. For example, the WHO recommends that only children aged over 12 years of age should be routinely vaccinated, while the Center for Disease Control and Prevention (CDC) in the United States recommends everyone ages 5 years and older receive a COVID-19 vaccine to help protect against COVID-19 [191,199]. More studies are required to assess the safety of these vaccines in young patients; some are still ongoing and are studying all age groups, including children younger than 5 years old [200,201]. Extreme caution is advised.

## 16. Conclusions

In conclusion, the clinical frame of COVID-19 infection is surely milder in children and adolescent populations, but this should not be a reason to underestimate the benefit of complete and safe immunization in these patients, which can be given by vaccines as they play a major role in the transmission of the infection. More evidence is needed to guide the vaccination campaign effectively and safely.

## Figures and Tables

**Table 1:** Summary of grading and clinical features of COVID-19 [151,152,153,154,155].

COVID-19 Severity	Symptoms
Asymptomatic grade	Positivity to the swabs/No clinical symptoms
Paucisymptomatic grade	Evidence of fever with/without asthenia. Absence of radiological and upper airway alterations evidence.
Moderate Grade	Presence of fever with/without fatigue. Alterations of the upper airway tract with cough or mild respiration distress. Evidence of inappetence with/without pneumonia could be observed by thorax RX of ultrasonography.
Severe Grade	Presence of fever accompanied by cough, SpO2 saturation < 92%, cyanosis, intermittent apnea, severe respiration distress, lethargy, convulsions, drowsiness and dehydration, high respiratory rate (RR): breaths/minute > 60 > 3 months; >50 > 3–12 months; >40 > 1–5 years; >30 > 5 years.
Critical Grade	Evidence of pediatric acute respiratory distress syndrome affecting multi-organ function. The clinical findings could be also accompanied by sepsis, septic shock, coma.

**Table 2:** COVID-19 therapies.

Author	Journal	Therapy	Protocols
Chiotos et al. [152]	J. Pediatric. Infect. Dis. Soc. 2020	(1) Remdesivir (2) Hydroxychloroquine (3) Lopinavir-Ritonavir	(1) Remdesivir: Body weight < 40 kg: Administration of 5 mg/kg at day 1 and 2.5 mg/kg/each treatment day. Body weight > 40 kg: Administration of 200 mg at day 1; and 100 mg each treatment day for almost 10 days, that could be reduced to 5 day in case of fast responders. (2) Hydroxychloroquine: Administration of 400 mg at the day 1 and 200 mg for 5 days. (3) Lopinavir and ritonavir Administration of 400 mg/ritonavir 100 mg in two doses/day for 7–14 days.
Maharaj et al. [153]	JAMA Pediatr. 2020	(1) Remdesivir; (2) Hydroxychloroquine	No antiviral effects of hydroxychloroquine due to the low plasma concentrations necessary against SARS-CoV-2.
Venturini et al. [37]	Ital. J. Pediatr. 2020	(1) Antipyretic (2) Antiviral drugs (3) Antibiotic (4) Steroid and antiviral drugs (5) Monoclonal antibodies	(1) Antipyretic therapy: Paracetamol for 10–15 mg/kg every 4–6 h in case of fever > 38 °C (2) Antiviral drugs: Avoid Lopinavir/Ritonavir and Hydroxychloroquine administration (3) Antibiotic: Empiric antibiotic administration is no recommended in severe and critical illness if bacterial infection is not present (4) Steroids: Moderate illness Dexamethasone (0.1–0.2 mg/kg) or methylprednisolone (1–2 mg/kg day) Remdesivir (5 mg/kg/1st day than 2.5 mg/kg for 5 days) Dexamethasone/methylprednisolone plus Remdesivir Severe illness Dexamethasone/methylprednisolone Dexamethasone/methylprednisolone plus Remdesivir (available for this group of patients only within clinical trials) Critical illness Dexamethasone/methylprednisolone (5) Monoclonal antibodies: Only with risk factors in mild cases

**Table 3:** Overview of COVID-19 lactoferrin supporting therapy.

Author	Journal	Therapy	Protocols
Lang et al. [160]	PloS ONE, 2011	(1) Lactoferrin (2) Heparin	Lactoferrin and heparin administration absolved a protective role for HEK293E/ACE-2 cells defense against SARS-CoV viral vector
Peroni et al. [154]	Acta Paediatr. 2020	(1) Lactoferrin	Lactoferrin demonstrates potential antiviral effects and protective action of the immunity system.

**Table 4:** Summary of adjuvant aerosol treatments for COVID-19.

Author	Journal	Therapy	Protocols
Parshuram et al. [171]	Crit. Care 2011	(1) Interferon alpha (IFN-α)	(1) Interferon alpha (IFN-α): inhalation (dose of 5 million units twice a day).
Chen et al. [100]	World J. Pediatr. 2020	(1) Interferon alpha (IFN-α)	(1) Interferon-α2b inhalation: Mild subjects: dose of 100,000–200,000 IU/kg twice a day for a total of 1 week. Severe subjects: 200,000–400,000 IU/kg twice a day for a total of 1 week.
Dong et al. [120]	Pediatrics 2020	(1) Interferon alpha (IFN-α)	(1) Interferon alpha (IFN-α): inhalation 2/3 times a day.

**Table 5:** COVID-19 antiviral dosing.

Drugs	Administration Protocols
Lopinavir Ritonavir	Subjects < 1 years old: Administration of 300 mg/75 mg/m^2^ twice/day Subjects of >1 years old and body weight < 15 kg: Administration of a dosage 12/3 mg twice/day; Subjects of >1 years old and body weight > 15 kg: Administration of a dosage 10/2.5 mg/kg twice/day
Remdesivir	Subjects with a body weight < 40 kg: 1st dose 5 mg/kg and 2.5 mg/kg/day for a total of 9 days.

**Table 6:** COVID-19 hydroxychloroquine dosage summary.

Drugs	Administration Protocols
Hydroxychloroquine	Administration of 6 mg/kg at day 1; and 3 mg/kg twice a day for a total of 5 days therapy

**Table 7:** COVID-19 immunomodulant dosage summary.

Drugs	Administration Protocols
Methylprednisolone	Subjects < 1 years old: Administration of 300 mg/75 mg/m^2^ twice/day Subjects of >1 years old and body weight < 15 kg: Administration of a dosage 12/3 mg twice/day; Subjects of >1 years old and body weight > 15 kg: Administration of a dosage 10/2.5 mg/kg twice/day
Anakinra	Subjects with a body weight < 40 kg: First dose endogenous administration of 5 mg/kg and 2.5 mg/kg/day for a total of 9 days.

**Table 8:** Basic features of the encountered randomized controlled trials that were designed for healthy children and adolescents.

Clinical Trial Patients	Sample Size	Follow-Up Duration	Study Design	Country	Reference
Subjects aged 3–17 years old	552 subjects	4.1 months	RCT Phase 1–2	China	Han et al., 2021 [197]
Subjects aged 12–15 years old with no positivity to the COVID-19	2264 subjects	4.7 months	RCT Phase 3	USA	Frenck et al., 2021 [198]

## Data Availability

Not applicable.

## Abbreviations

ACE-2	Angiotensin-converting enzyme 2
ARDS	Acute Respiratory Distress Syndrome
AUC	Area under the ROC Curve
CAR T cells	Chimeric antigen receptor T cells
CDC	Center for Disease Control and Prevention
COVID-19	Coronavirus Disease 2019
ECG	Electrocardiogram
EV	Endogenous
G6PDH	Assay glucose-6-phosphate dehydrogenase
HIV	Human immunodeficiency virus
HSPG	Heparan sulphate proteoglycans
IFN-α	Interferon alpha
IL-6	Interleukin 6
ISS	Istituto Superiore di Sanità
IV	Intravenous
MERS-CoV	Middle East Respiratory Syndrome Coronavirus
MSCs	Mesenchymal stem cell
MIS-C	Multisystem inflammatory syndrome
NIV	Non-invasive ventilation
MyD88	Myeloid differentiation primary response 88
PARSD	Pediatric acute respiratory distress syndrome
PEWS	Pediatric Early Warning Score
RBD-IgG	Receptor-binding domain
ROC	Receiver Operator Characteristic
RR	Respiratory rate
RSV	Respiratory Syncytial Virus
SARS-CoV-2	Severe Acute Respiratory Syndrome Coronavirus 2
SiTIP	Italian Society of Pediatric Infectious Diseases
SpO2	Oxygen saturation
VOC	Variant of concern
WHO	World Health Organization
